# Early Dynamics of Cerebrospinal CD14+ Monocytes and CD15+ Granulocytes in Patients after Severe Traumatic Brain Injury: A Cohort Study

**DOI:** 10.1155/2015/197150

**Published:** 2015-10-19

**Authors:** Lukas Kurt Postl, Viktoria Bogner, Martijn van Griensven, Marc Beirer, Karl Georg Kanz, Christoph Egginger, Markus Schmitt-Sody, Peter Biberthaler, Chlodwig Kirchhoff

**Affiliations:** ^1^Department of Trauma Surgery, Klinikum rechts der Isar, Technische Universitaet Muenchen, 81675 Munich, Germany; ^2^Department of Trauma Surgery, Ludwig Maximilians University Munich, 80336 Munich, Germany; ^3^Department of Trauma Surgery, Klinikum Passau, 94032 Passau, Germany; ^4^Department of Orthopedic Surgery, Ludwig Maximilians University Munich, 81377 Munich, Germany

## Abstract

In traumatic brain injury (TBI) the analysis of neuroinflammatory mechanisms gained increasing interest. In this context certain immunocompetent cells might play an important role. Interestingly, in the actual literature there exist only a few studies focusing on the role of monocytes and granulocytes in TBI patients. In this regard it has recently reported that the choroid plexus represents an early, selective barrier for leukocytes after brain injury. Therefore the aim of this study was to evaluate the very early dynamics of CD14+ monocytes and CD15+ granulocyte in CSF of patients following severe TBI with regard to the integrity of the BBB. Cytometric flow analysis was performed to analyze the CD14+ monocyte and CD15+ granulocyte population in CSF of TBI patients. The ratio of CSF and serum albumin as a measure for the BBB's integrity was assessed in parallel. CSF samples of patients receiving lumbar puncture for elective surgery were obtained as controls. Overall 15 patients following severe TBI were enrolled. 10 patients were examined as controls. In patients, the monocyte population as well as the granulocyte population was significantly increased within 72 hours after TBI. The BBB's integrity did not have a significant influence on the cell count in the CSF.

## 1. Introduction

Traumatic brain injury (TBI) is especially prevalent in young adults [1] and represents one of the leading causes of death and of persistent damage of neurocognitive functions. The outcome is primarily determined by the initial trauma resulting from the physical impact and secondarily determined by the extent of secondary injury to the brain in terms of brain edema, increased intracranial pressure, and delayed cell destruction [2]. These secondary injury mechanisms could be responsible for the development of neurological deficits after TBI evolving minutes to days or even months after the primary mechanical injury [3]. The delayed incidence of the secondary injury mechanisms indicates that there might be a time window for therapeutic interventions to reduce brain tissue damage and improve the functional neurological outcome [3]. Therefore improved understanding of the complex processes following TBI [3] is crucial for the development of an effective neuroprotective treatment. Although the key role of the systemic cellular immune response in patients following multiple trauma has been emphasized by several authors, there is only a limited number of studies analyzing the cellular response of the key inflammatory cells such as monocytes and granulocytes in the cerebrospinal fluid (CSF) of patients following TBI [4–6]. Monocytes are characterized by CD14, a 56 kDa cell membrane anchored protein [7, 8]. In parallel, the carbohydrate antigen CD15 (the carbohydrate antigen 3-fucosyl-N-acetyl-lactosamine) with an approximate molecular mass of 165 and 105 kDa is expressed on membrane glycoproteins of neutrophil granulocytes [9, 10]. Under physiologic conditions the CSF is separated from peripheral and cerebral blood flow by the blood brain barrier (BBB). In analyzing the dynamics of monocytes and granulocytes in CSF of patients after TBI, the question arises whether potential changes of cellular contents occur due to a disrupted BBB or by a certain mechanism still to be explained. It is well known that the leukocyte count of the CSF is far lower compared to peripheral blood. Therefore a disrupted BBB potentially leads to an increase of leukocytes in the CSF following cell leakage due to disrupted blood vessels.

Therefore the aim of the present study was to evaluate the fraction of CD14+ monocytes and CD15+ granulocytes in CSF of patients following TBI beginning at the time of admission until 72 hours (hrs) after TBI. The influence of the BBB integrity on the number of monocytes and granulocytes in CSF was also assessed in this context.

## 2. Patients and Methods

### 2.1. Study Design and Patient Collective

The study protocol was approved by the university's board of ethics (reference number 330/03). Inclusion criteria for prospective enrolment were presence of isolated closed TBI, initial Glasgow Coma Score (GCS) ≤ 8 points (i.e., severe brain injury), proof of intracranial bleeding (ICB) on the initial cranial computed tomography scan (CCT; performed within 90 minutes after TBI), and the indication for placing an external ventricular drainage (EVD) catheter. Exclusion criteria were a history of preexisting neurological, malignant, or chronic inflammatory disease. Written informed consent was obtained when the patient regained consciousness. In case of remaining unconscious, a next of kin or a legal representative was asked for the presumed consent.

### 2.2. Clinical and Surgical Procedures

An external ventricular drainage (EVD) catheter (TraumaCath, Integra Neurosciences, Plainsboro, USA) was placed in the frontal horn of the lateral ventricle using CT fluoroscopy guidance to continuously monitor the intracranial pressure (ICP) and to drain CSF [11]. After a CT scan performed to control the correct placement of the drainage, the patients were referred to the intensive care unit (ICU) and treated according to the guidelines of the Brain Trauma Foundation [12]. If the ICP remained under 15 mmHg for at least 72 hrs without mannitol administration or CSF drainage, the EVD was removed.

### 2.3. Sampling Procedures

The first sampling took place immediately after the insertion of the EVD (90 ± 45 minutes after admission to the hospital). Further samples were obtained 12, 24, 48, and 72 hrs after TBI. At every sampling time point 4 mL of drained CSF, 5 mL of peripheral serum blood, and 5 mL of EDTA blood were collected. 500 *μ*L of CSF was sent to the institute of laboratory medicine of our university hospital for the determination of the total cell counts and albumin levels in CSF. The serum blood was used for the determination of the albumin levels in peripheral blood by the institute of laboratory medicine. Albumin levels were necessary for the evaluation of the blood brain barrier (BBB). The EDTA blood was sent to the institute of laboratory medicine and was used for peripheral differential blood counts.

### 2.4. Fluorescence Labeling

For the fluorescence activated cell sorting (FACS) analysis, 3 mL of the cerebrospinal fluid samples was centrifuged with 1200 G at 4°C for 10 minutes to remove the supernatant and processed immediately. The cell pellet was resuspended in 200 *μ*L FC buffer suspension (0.01 M phosphate buffered saline, pH 7.2, 1% bovine serum albumin, and 5% fetal calf serum (FCS; 0.02% NaN_3_)).

20 *μ*L of the resuspension was incubated for 20 minutes with 3 *μ*L fluorochrome-labeled antibodies in the dark on ice. All antibodies CD14 TC and CD15 FITC (Caltag Laboratories, Hamburg, Germany) were used in single-stain arrays. For lysing of the erythrocytes, 500 *μ*L FACS lysing solution (Caltag Laboratories, Hamburg, Germany) was used and afterwards the suspension was incubated for another 10 minutes. After washing with 500 *μ*L PBS (5% fetal calf serum (FCS; 0.02% NaN_3_, Pettenkofer, Munich, Germany)) twice, the suspension was centrifuged again for 10 minutes with 1200 rounds per minute at 4°C.

### 2.5. Flow Cytometric Analysis

The FACS analysis was performed using a four-channel Epics XL MCL flow cytometer (Beckman-Coulter, Krefeld, Germany) with an air-cooled 15 mW 488 nm argon ion laser. A 530 nm filter for FITC (fluorescein isothiocyanate) and a >650 nm filter for TC (Tri-Color, Caltag Laboratories, Hamburg, Germany) were applied. Data acquisition was obtained using the Expo 32 Software (Beckman-Coulter, Krefeld, Germany). Spectral compensations and instrument settings were appropriately adjusted. Fluidics and Instruments were regularly checked using FlowCheck beads (Beckman-Coulter, Krefeld, Germany). For a respective analysis, the cytometer counted 5000 events (cells) in a list mode. The free WinMDI Software (Version 2.8, Bio-Soft Net [13]) was used for data analysis. Antibody-positive cells were determined as the most positive on side scatter versus TC (CD14) and FITC (CD15) positive cells, respectively. Antibody-positive cells were counted as percentage of the total cell count.

### 2.6. Assessment of the Blood Brain Barrier Function

In order to determine whether CD14+ monocytes' and CD15+ granulocytes' dynamics correlate to posttraumatic BBB disruption, the ratio of CSF and serum albumin (*Q*_alb_) was calculated at each sampling time point. Albumin levels were evaluated by using standardized turbidimetric assays (Cobas Integra Albumin, Roche Diagnostics, Mannheim, Germany). According to Reiber and Felgenhauer, *Q*_alb_ is a sensitive parameter for the analysis of the BBB [14]. In this study, the disturbance of the BBB was assessed as follows: *Q*_alb_ values below 0.007 were considered as normal, values between 0.007 and 0.01 as mild dysfunction, values between 0.01 and 0.02 as moderate, and levels above 0.02 as severe dysfunction of the BBB. Normal and mild dysfunction were defined as intact BBB whereas moderate and severe dysfunction were defined as disrupted BBB. To assess the influence of the BBB's function on the dynamics of the monocytes, the enrolled patients were divided into two groups: group I presenting with an intact BBB and group II with a defect BBB.

### 2.7. Data Analysis

Data are given in mean ± standard error of the mean (SEM). For comparing patient values with control values or the patient value at the time of admission, the data was analyzed using ANOVA (analysis of variance on ranks) for nonparametric data according to Kruskal-Wallis. For analysis of significant differences over the course of time ANOVA was performed using the Student-Newman-Keuls test. For determination of specific differences Dunn's test was performed. The level of significance was set to ≤0.05. For all statistical analysis the Sigma Stat 3.0 software package (SPSS Inc., Chicago, USA) was used.

## 3. Results

### 3.1. Patients and Demographic Data

In total, 23 patients with isolated severe TBI were enrolled (8 females and 15 males; age 46 ± 5 years). Eight patients died within 24 hrs after TBI, because of therapy resistant brain swelling and/or brain stem herniation and thus had to be excluded. CSF was obtained from 10 control patients (5 females and 5 males; age 44 ± 9 years) who underwent spinal anesthesia for an elective orthopedic procedure of the lower extremity.

### 3.2. Dynamics of CD14+ Monocytes

**Figure 1:**
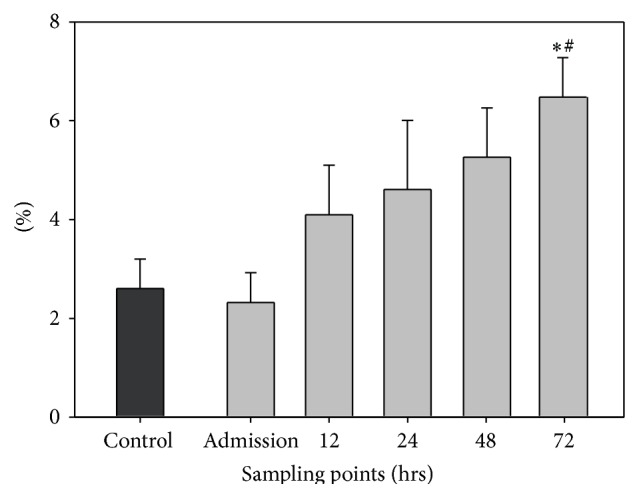
Dynamics of CD14+ monocytes. The graph demonstrates the number of CD14+ monocytes in percent (%) of all CSF cells at the different sampling time points. Data are given in mean ± standard error of the mean. ^*∗*^ *p* < 0.05 versus control group; ^#^ *p* < 0.05 versus admission.

The baseline value of the control group monocyte population was 2.6 ± 0.6%. At admission the monocyte count was 2.32 ± 0.6% in the TBI patient group, not significantly different to the control group. The monocyte population increased to 4.10 ± 1.0% at 12 hrs, to 4.61 ± 1.4 % at 24 hrs, and to 5.26 ± 1.0% at 48 hrs. Although a continuous increase was observed, there was no statistically significant difference in comparison to the admission level. In addition, the monocyte populations at 12, 24, and 48 hrs were not significantly different in comparison to the baseline level of healthy controls. However, 72 hrs after TBI the monocyte population accounted for 6.48 ± 0.8% and was thereby significantly higher in comparison to the value at admission. Furthermore the monocyte population was significantly higher in comparison to the baseline level of the healthy control group (see Figure 1).

#### 3.2.1. Group I (Intact BBB, *n* = 9)

At admission the CD14+ monocyte count of patients with intact BBB was 2.61 ± 0.9%, which was not significantly different in comparison to the baseline level of the control group. The fraction increased to 4 ± 1.5% and 4.34 ± 1.4% at 12 hrs and 24 hrs after TBI, respectively. The cell counts were still not significantly different in comparison to the cell level at admission. In addition, there was no significant difference in comparison to the baseline level of the control group. At 48 hrs after TBI the population accounted for 6.40 ± 1.5%, which was significantly higher than the baseline level of the control group (*p* < 0.05). At 72 hrs the CD14+ monocytes further increased to a value of 6.91 ± 1.0%. This was significantly higher than the baseline value of the control group and significantly elevated compared to the admission value.

#### 3.2.2. Group II (Disrupted BBB, *n* = 6)

At the time of admission the monocyte population accounted for 1.89 ± 0.9 and it was only slightly but not significantly higher at 12 hrs after TBI (4.25 ± 1.6%). At 24 hrs the population dropped to a level of 3.53 ± 1.1% and there was still no significance compared to the value at the time of admission (0 hrs). In addition, the monocyte population at the time of admission, 12 hrs, and 24 hrs after TBI was not significantly different in comparison to the baseline level of the control group. At 48 hrs and at 72 hrs after TBI the populations were 5.02 ± 3.2% and 5.83 ± 1.4%, respectively; both values were significantly higher than the value at the time of admission as well as the baseline level of the control group.

#### 3.2.3. BBB Evaluation

**Figure 2:**
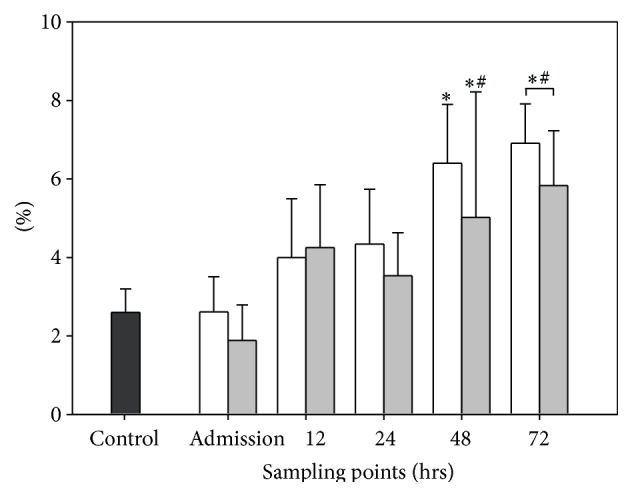
Dynamics of CD14+ monocytes and function of the blood brain barrier. The graph demonstrates the number of CD14+ monocytes in percent (%) of all CSF cells. Graphs in white demonstrate data of patients with intact blood cerebrospinal fluid barrier (*n* = 9), whereas graphs in grey demonstrate data of patients with disrupted blood cerebrospinal fluid barrier (*n* = 6). Data are given in mean ± standard error of the mean. ^*∗*^ *p* < 0.05 versus control group; ^#^ *p* < 0.05 versus admission.

Regarding the evaluation of the BBB there were no significant differences between group I with intact BBB and group II with disrupted BBB at the defined sample points (0, 12, 24, 48, and 72 hrs after TBI; see Figure 2).

### 3.3. Dynamics of CD15+ Granulocytes

**Figure 3:**
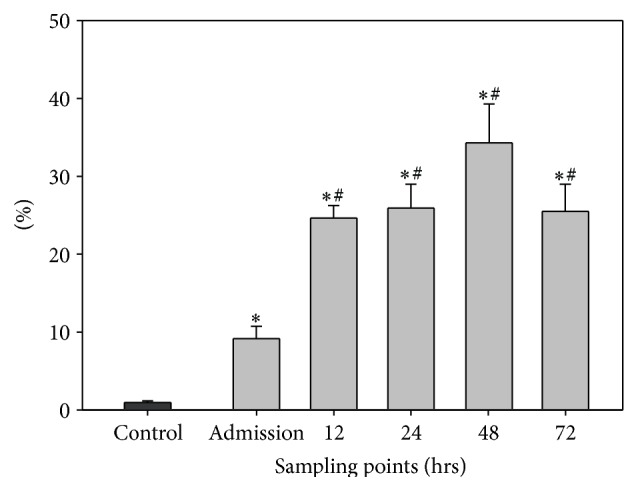
Dynamics of CD15+ granulocytes. The graph demonstrates the number of CD15+ granulocytes in percent (%) of all CSF cells at the different sampling points. Data are given in mean ± standard error of the mean. ^*∗*^ *p* < 0.05 versus control group; ^#^ *p* < 0.05 versus admission.

The control group value accounted for 0.98 ± 0.2%. The population of the CD15+ granulocytes was already significantly elevated at admission (9.15 ± 1.6%) compared to the baseline level of controls. At 12 hrs the value increased to 24.65 ± 1.6% and was therefore significantly higher in comparison to the admission time point. The granulocyte populations were 25.9 ± 3.1, 34.3 ± 5.0, and 25.5 ± 3.5 at 24, 48, and 72 hrs, respectively, so that the values remained significantly elevated compared to the admission levels as well as the baseline level of controls (see Figure 3).

#### 3.3.1. Group I (Intact BBB, *n* = 9)

The population of granulocytes was already significantly elevated (8.61 ± 1.3%) at admission compared to the baseline level of the control group (0.98 ± 0.2%). At 12 hrs after TBI the population accounted for 26.96 ± 2.6%, which was significantly higher in comparison to admission. The granulocytes were 25.42 ± 4.6%, 33.38 ± 7.0%, and 22.27 ± 4.6% at 24 hrs, 48 hrs, and 72 hrs, respectively. These populations remained significantly higher in comparison to admission. In addition the granulocytes in group I were significantly higher compared to the baseline level of the control group at every sampling time point.

#### 3.3.2. Group II (Disrupted BBB, *n* = 6)

The population of granulocytes accounted for 14.92 ± 1.0% at admission and was therefore significantly higher in comparison to the control group (0.98 ± 0.2%). At 12 hrs and at 48 hrs the values increased to 24.48 ± 1.3% and 28.37 ± 2.9%, respectively. At 48 hrs granulocytes increased to 35.80 ± 7.6% and were therefore significantly higher in comparison to admission. At 72 hrs granulocytes dropped to 22.27 ± 4.6%, so that there was no further significant difference in comparison to admission levels. The populations of granulocytes in group II were significantly higher in comparison to the baseline level of the control group for every sampling time point.

#### 3.3.3. BBB Evaluation

**Figure 4:**
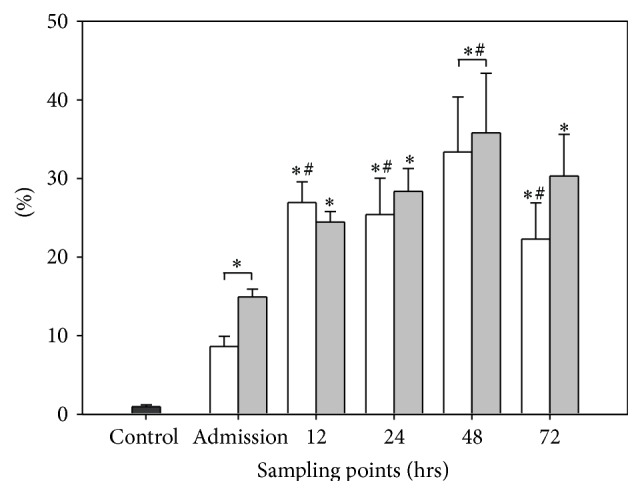
Dynamics of CD15+ granulocytes and function of the blood brain barrier. The graph demonstrates the number of CD15+ granulocytes in percent (%) of all CSF cells. Graphs in white demonstrate data of patients with intact blood cerebrospinal fluid barrier (*n* = 9), whereas graphs in grey demonstrate data of patients with disrupted blood cerebrospinal fluid barrier (*n* = 6). Data are given in mean ± standard error of the mean. ^*∗*^ *p* < 0.05 versus control group; ^#^ *p* < 0.05 versus admission.

Regarding the evaluation of the BBB there was no significant difference between group I with intact BBB and group II with disrupted BBB (see Figure 4).

### 3.4. CSF Total Cell Count

The absolute level of cells (total cell count) as well as the percentages of monocytes and granulocytes at the various sample points is given in Tables 1, 2, and 3. There was no significant difference found comparing the sampling points to base line levels at admission. There was also no difference comparing patients with intact and disrupted BBB.

### 3.5. Peripheral Blood Monocyte and Granulocyte Count

The populations of monocytes and granulocytes in peripheral blood are provided in Table 4. There were no significant changes over the course of time.

## 4. Discussion

In the present study the dynamics of CD14+ monocytes and CD15+ granulocytes in the cerebrospinal fluid of patients suffering from traumatic brain injury were evaluated in the direct posttraumatic course over 72 hours for the first time. CD14+ monocyte and CD15+ granulocyte cell populations were significantly elevated within the first 72 hrs after TBI compared to baseline levels at admission to hospitals as well as in comparison to healthy controls. Regarding a potential dysfunction of the BBB no significant difference was found between TBI patients with intact BBB and TBI patients with disrupted BBB, respectively.

Only patients with isolated TBI were included to avoid an increase of monocytes and granulocytes in the peripheral blood not resulting from TBI and a potential consecutive bias. In addition the inclusion criteria in terms of severe TBI (GCS ≤ 8) and signs of intracerebral hemorrhage were established to ensure that an adequate trauma to the brain had happened possibly resulting in detectable and significant changes of the CSF on cellular level. In conclusion the inclusion criteria lead to enrollment of patients in critical conditions, so that research as conducted in this study should form the basis of future research regarding especially a potential modification of therapeutic options after TBI to avoid secondary brain damage.

In general the effect of moderate (and even mild) TBI on the dynamics of monocytes and granulocytes would be additionally interesting for the understanding of TBI and the processes happening in the brain on cellular level. However harvest of CSF is quite difficult to account for in patients who are not considered to get an EVD placed. In the current literature several studies report detection of pro- and anti-inflammatory mediators such as cytokines or interleukins in the CSF of patients [15]. However, there is also evidence in the literature that secondary brain damage may also develop without an increase of interleukins in CSF [16]. In this context a functional involvement of immunocompetent cells like monocytes [17–21] and granulocytes [22, 23] in the formation of secondary brain damage was described.

In the present study the population of CD14+ monocytes and CD15+ granulocytes increased significantly within the first 72 hrs after TBI compared to the corresponding populations of the control group and even to the cell populations at the time of admission. Consecutively the question arose whether this was due to a nonmodulated influx of these cells from the peripheral blood into CSF because of a disrupted BBB or whether the cells were transported across the BBB via certain mechanisms to be described. In this context the presented results demonstrate that the integrity of the BBB had no significant influence on the populations of the CD14+ monocytes and CD15+ granulocytes in the time course of the first 72 hours after TBI. In addition there was no significant drop of monocyte or granulocyte populations detected in the peripheral blood. Therefore our data does not support the theory of a nonmodulated influx of cells from the peripheral blood. However, subdividing into groups with a rather small number of *n* < 10 has to be critically interpreted from the statistical point of view. In this context recent publications demonstrated a monocyte trafficking across the BBB using a transmission electron microscopy in a rat TBI model [24, 25]. In this regard Schwartz and Baruch recently reported that the choroid plexus represents an early entry site for leukocytes after injury of the CNS (Central Nervous System) [26]. Shechter et al. also recently found that monocytes primarily traffic through the choroid plexus after CNS injury [27]. These recently reported findings possibly help to explain the significantly elevated populations of CD14+ monocytes and CD15+ granulocytes in the CSF in the early phase of TBI patients in the present study.

The CD15+ granulocytes were already significantly and nearly tenfold increased at the time of admission indicating a very fast response of the quantity of granulocytes after TBI. In addition the granulocyte population further increased; 12 hrs after TBI the population was already significantly higher than at the time of admission. These data show that the granulocytes are fast-responder and available for migration into the CSF in sufficient numbers. Also the data about the dynamics of the cells provide initial evidence that the granulocytes could play an important role in the very early posttraumatic phase after severe TBI.

The increase of CD14+ monocytes did not lead to a significantly higher level of the monocyte population until 72 hrs after TBI. The comparably slower increase of monocytes could mean that these cells are not as much involved in the early response after TBI as granulocytes. This could however just indicate that monocytes are far more immobile than granulocytes or just not in an equal number available for migration into the CSF. In this regard it would be very interesting to determine the population of activated cells among the overall CD14+ monocytes. Future work is therefore necessary to further understand the role of monocytes in TBI.

A limitation of this study to be mentioned is that only dynamics of CD14+ monocytes and CD15+ granulocytes were analyzed and that the distinct cause of the resulting increase was not determined. In this regard it has to be stated that the placement of an EVD itself can be considered as some kind of brain injury. Hence, the inflammatory response in the CSF could potentially be caused by placement of the EVD. This is a potential bias, which is not excluded by the control CSF group. In the control group CSF was obtained via lumbar puncture for spinal anesthesia which is a completely different injury model concerning the CSF collection. However our study was initiated to measure the effect of immunocompetent cells in TBI in the early posttraumatic phase. Strictly speaking this study compared the inflammation response after two known TBI events (the first event(s) up to 90 minutes previous to the second TBI event considering placement of the EVD as TBI event) to a control group with a minor spinal injury in terms of spinal anesthesia. This should be kept in mind when interpreting the results. Therefore a further limitation of the study is that a second control group with patients needing EVD or a ventricular shunt for other reasons than TBI (e.g., hydrocephalus) could have also been included possibly allowing for recognition of potential effects caused by placing an EVD. We were not able to provide data from such appropriate control patients in our academic university trauma department. Therefore a multicenter study should be considered for future investigation. However this study analyzed the dynamics of monocytes and granulocytes after severe TBI events which was an important first step that future research can build on.

In conclusion the populations of CD14+ monocytes and CD15+ granulocytes significantly increased compared to the cell populations of the control group and even to the cell populations at the time of admission in the patient group, but it remained unclear how this increase occurred and whether the increase influenced the outcome of TBI patients. Further research is necessary and is the focus of our study group's current work.

## Figures and Tables

**Table 1:** Total cell count in the CSF of patients.

Cells/*µ*L	Admission	12 hrs	24 hrs	48 hrs	72 hrs
All patients (*n* = 15)	30.7 ± 5.6	17.8 ± 2.5	16.9 ± 3.0	24.6 ± 4.2	27.8 ± 4.9
Intact BBB (*n* = 9)	31.4 ± 7.8	12.75 ± 1.8	11.0 ± 1.2	17.5 ± 2.3	25.4 ± 5.9
Disrupted BBB (*n* = 6)	29.8 ± 8.7	24.5 ± 4.1	24.7 ± 5.9	34.2 ± 8.5	31.0 ± 9.0

Total numbers of CSF cells. Data given as mean ± SEM.

BCSFB: blood cerebrospinal fluid barrier.

CSF: cerebrospinal fluid.

TBI: traumatic brain injury.

**Table 2:** Monocyte count in the CSF of patients.

Monocyte count (%)	Admission	12 hrs	24 hrs	48 hrs	72 hrs
All patients (*n* = 15)	5.3 ± 0.4	6.9 ± 0.6	6.3 ± 0.4	7.6 ± 0.9	5.7 ± 0.5
Intact BBB (*n* = 9)	5.6 ± 0.6	6.7 ± 0.7	6.1 ± 0.5	7.4 ± 0.7	5.9 ± 0.76
Disrupted BBB (*n* = 6)	4.9 ± 0.4	7.4 ± 0.9	6.6 ± 0.6	7.8 ± 1.8	5.2 ± 0.7

Percentages of monocytes. Data given as mean ± SEM.

BCSFB: blood cerebrospinal fluid barrier.

CSF: cerebrospinal fluid.

TBI: traumatic brain injury.

**Table 3:** Granulocyte count in the CSF of patients.

Granulocyte count [%]	Admission	12 hrs	24 hrs	48 hrs	72 hrs
All patients (*n* = 15)	63.9 ± 3.7	69.9 ± 3.2	70.6 ± 3.4	66.2 ± 5.5	65.9 ± 3.6
Intact BBB (*n* = 9)	63.9 ± 4.1	71.4 ± 2.8	71.9 ± 3.2	64.7 ± 8.4	64.4 ± 4.3
Disrupted BBB (*n* = 6)	64 ± 6.0	67.7 ± 6.1	68.5 ± 6.4	68.5 ± 5.1	68 ± 5.2

Percentages of granulocytes. Data given as mean ± SEM.

BCSFB: blood cerebrospinal fluid barrier.

CSF: cerebrospinal fluid.

TBI: traumatic brain injury.

**Table 4:** Peripheral blood monocytes and granulocytes.

Cells [%]	Admission	12 hrs	24 hrs	48 hrs	72 hrs
Monocytes (*n* = 15)	5.3 ± 0.4	7.0 ± 0.6	6.3 ± 0.4	7.6 ± 1.0	5.7 ± 0.5
Intact BBB (*n* = 9)	5.6 ± 0.6	6.7 ± 0.8	6.1 ± 0.6	7.4 ± 0.8	6.0 ± 0.7
Disrupted BBB (*n* = 6)	4.9 ± 0.5	7.4 ± 1.1	6.6 ± 0.7	7.8 ± 2.2	5.2 ± 0.8

Granulocytes (*n* = 15)	64.0 ± 3.7	69.9 ± 3.2	70.6 ± 3.4	70.9 ± 2.9	65.9 ± 3.6
Intact BBB (*n* = 9)	64.0 ± 4.8	71.4 ± 3.2	71.9 ± 3.6	72.5 ± 3.4	64.4 ± 4.9
Disrupted BBB (*n* = 6)	64.0 ± 6.6	67.7 ± 6.7	68.5 ± 7.0	68.5 ± 5.5	68 ± 5.7

Percentages of cells. Data given as mean ± SEM.

BCSFB: blood cerebrospinal fluid barrier.

TBI: traumatic brain injury.
